# *Panax notoginseng saponins* alleviate alveolar bone loss and reprogram macrophages in diabetic periodontitis rats

**DOI:** 10.1590/1678-7757-2025-0424

**Published:** 2025-10-20

**Authors:** Xue-wei JIANG, ZHU Jun, Wen CHENG, FAN De-sheng, Lei ZHEN

**Affiliations:** 1 Shanghai Jiao Tong University School of Medicine Tongren Hospital Department of Stomatology Shanghai China Shanghai Jiao Tong University School of Medicine, Tongren Hospital, Department of Stomatology, Shanghai, China.; 2 Tongji University, School of Medicine Tongji Hospital Department of Stomatology Shanghai China Tongji University, School of Medicine, Tongji Hospital, Department of Stomatology, Shanghai, China.; 3 Baoshan Hospital Shanghai University of Traditional Chinese Medicine Department of Medical Equipment Shanghai China Baoshan Hospital Affiliated to Shanghai University of Traditional Chinese Medicine, Department of Medical Equipment, Shanghai, China.; 4 Baoshan Hospital Shanghai University of Traditional Chinese Medicine Department of Pathology Shanghai China Baoshan Hospital Affiliated to Shanghai University of Traditional Chinese Medicine, Department of Pathology, Shanghai, China.

**Keywords:** Diabetes, Periodontitis, PNS, Alveolar bone loss, Macrophages

## Abstract

Diabetic periodontitis (DP) features accelerated alveolar bone destruction driven by impaired immune regulation and compromised bone metabolism.

**Objective:**

This study investigated the therapeutic potential of *Panax notoginseng saponins* (PNS) against alveolar bone loss in a rat DP model and elucidated its mechanisms of action.

**Methodology:**

Male Sprague-Dawley rats were allocated to four groups: control, periodontitis control (CP), diabetic periodontitis (DP), and DP + PNS (80 mg/kg/day). Diabetes was induced by streptozotocin injection, followed by ligature-induced periodontitis at the maxillary first molar. After 4 weeks of PNS treatment, alveolar bone samples were analyzed by micro-CT, histomorphometry, immunohistochemistry, and immunofluorescence.

**Results:**

Micro-CT and H&E analyses revealed severe alveolar bone resorption in DP rats, whereas PNS treatment substantially mitigated these destructive changes. TRAP staining demonstrated that PNS significantly suppressed osteoclast formation and activity. Immunohistochemistry detected upregulated expression of OCN in PNS-treated groups, indicating enhanced osteogenic differentiation. Immunofluorescence analysis showed that PNS promoted a phenotypic shift in macrophages, reducing pro-inflammatory M1 polarization (iNOS+) while increasing anti-inflammatory M2 populations (Arg-1+). This shift correlated with decreased interleukin-6 (IL-6) and elevated interleukin-10 (IL-10) levels.

**Conclusions:**

PNS attenuates alveolar bone loss in diabetic periodontitis by inhibiting osteoclastogenesis, stimulating osteogenic activity, and modulating macrophage polarization toward an anti-inflammatory M2 phenotype. These actions collectively reduce inflammation and promote tissue regeneration, highlighting PNS as a promising candidate for managing DP-related bone destruction.

## Introduction

Diabetic periodontitis (DP), a bidirectional complication arising from diabetes mellitus (DM) and periodontal disease,^1,2^ represents a critical unmet clinical challenge in modern dentistry.^3,4^ This condition is driven by a self-perpetuating cycle of hyperglycemia-induced tissue damage, dysbiotic microbial communities, and dysregulated immune-inflammatory responses, ultimately resulting in accelerated alveolar bone resorption, periodontal ligament degradation, and eventual tooth loss. Patients with DM exhibit heightened susceptibility to periodontal destruction due to hyperglycemia-aggravated inflammation and compromised immune defenses.^5,6^ Moreover, periodontal inflammation exacerbates systemic metabolic dysregulation, as pro-inflammatory mediators released into circulation impair glycemic control, thereby worsening diabetic complications.^7-9^ Consequently, effective management of DP is crucial not only for maintaining oral health but also for mitigating the systemic inflammatory burden that amplifies diabetes-related pathologies. Current treatments for periodontitis include subgingival scaling and root planing, periodontal surgical interventions, and adjunctive drug therapy. However, the effects of these treatments on DP are limited and frequently ineffective due to excessive oxidative stress activation, persistent inflammation, abnormal immune regulation, and impaired mesenchymal stem cell (MSCs) function under diabetic conditions.

Emerging evidence highlights macrophages as master regulators of periodontal tissue dynamics through their metabolic and phenotypic plasticity.^10-12^These sentinel immune cells exist along a functional spectrum ranging from pro-inflammatory M1-polarized states (characterized by iNOS/CD86 expression and IL-6/TNF-α secretion) to tissue-reparative M2 phenotypes (marked by Arg-1/CD206 expression and IL-10/TGF-β production). In diabetic microenvironments, chronic hyperglycemia induces metabolic reprogramming that skews macrophage polarization toward destructive M1 dominance.^13-15^ This pathological shift creates an osteoclastogenic niche through dual mechanisms: direct stimulation of osteoclast precursors via RANKL/IL-6 signaling^16^ and indirect suppression of osteoblast differentiation through Wnt/β-catenin pathway inhibition.^17^The resultant imbalance in bone remodeling, coupled with impaired angiogenesis and collagen deposition, drives progressive periodontal breakdown. Thus, therapeutic strategies capable of restoring macrophage polarization balance may fundamentally disrupt the pathophysiological cascade of DP.

*Panax notoginseng* (Burk.) F.H. Chen, a revered herb in traditional Chinese medicine, has drawn increasing attention for its bioactive saponin constituents. *Panax notoginseng saponins* (PNS), the major pharmacological components—including ginsenosides Rg1, Rb1, and notoginsenoside R1—exhibit pleiotropic effects on inflammation, angiogenesis, and bone metabolism.^18-20^Pre-clinical investigations have demonstrated that PNS can inhibit LPS-induced NF-κB activation in macrophages, reducing the production of pro-inflammatory cytokines while enhancing anti-inflammatory mediator secretion.^21^ In osteoporotic models, PNS promotes osteoblast proliferation via the BMP-2/Smads and Wnt/β-catenin pathways and suppresses osteoclast differentiation by downregulating RANKL-induced c-Fos and NFATc1 expression.^22,23^ Notably, in rheumatoid arthritis models, PNS has been shown to reprogram macrophage polarization from M1 to M2 phenotypes, attenuating synovial inflammation and cartilage degradation through modulation of the JAK/STAT and PI3K/AKT signaling pathways.^24^ These findings suggest that PNS may exert protective effects in DP by targeting both immune dysregulation and bone remodeling dysfunction.

Despite these pharmacological insights, the therapeutic potential of PNS in diabetic periodontitis remains unexplored. Therefore, this study investigates the alveolar bone-protective effects of PNS in a rat model of diabetic periodontitis, with particular emphasis on its capacity to reprogram macrophage polarization states. By elucidating the role of PNS in modulating macrophage-mediated bone homeostasis, our findings may provide novel insights into DP treatment and identify PNS as a potential therapeutic agent for this challenging diabetic complication.

## Methodology

### Animal model establishment

All experimental procedures were approved by the Animal Ethics Committee of Tongji Hospital (Approval Number: 2024-DW-012) and complied with institutional guidelines for animal care and use. Sample size was estimated using the G*Power analysis software program with α=0.05 and 1-β (power)=0.95. Considering potential losses during the experiment, six rats were allocated per group. Animals were randomly assigned to each group using a computer-generated random number table. All data analyses were performed by an evaluator blinded to group allocation to minimize bias.

A total of 24 male Sprague-Dawley (SD) rats (6 weeks old; weight 200–220 g) were obtained from SLAC Laboratory Animal Co. Ltd. (Shanghai, China). Following a one-week acclimatization period, diabetes was induced via a single intraperitoneal injection of streptozotocin (STZ; Sigma-Aldrich, St. Louis, MO, USA), dissolved in 0.1 mol/L citrate buffer (Sigma), which was administered at a dose of 65 mg/kg. Rats with fasting glucose levels ≥16.7 mmol/L at 72 h after injection were defined as diabetic. One week after diabetes confirmation, diabetic periodontitis (DP) was induced by ligating the bilateral maxillary first molars with 0.2 mm diameter stainless-steel orthodontic wire for 4 weeks to facilitate periodontal inflammation. Non-diabetic rats subjected to identical ligation served as the periodontitis control group (CP). Diabetic rats with ligation were further divided into two subgroups: the untreated diabetic periodontitis group (DP) and diabetic periodontitis rats treated with *Panax notoginseng saponins* (PNS) (DP+PNS). The DP+PNS group received PNS (80 mg/kg/day), dissolved in corn oil, administered by oral gavage once daily for 4 weeks concurrent with the ligation period.

### Micro-CT analysis

At the end of the experiment, the maxilla was dissected free from surrounding soft tissues. Samples were fixed in 4% paraformaldehyde (Beyotime Biotechnology, Shanghai, China) and analyzed using a micro-CT system. Bone loss in the first molars of each rat was measured by estimating the vertical distance from the alveolar bone crest to the cementoenamel junction at six sites: mesiobuccal, midbuccal, distobuccal, mesiopalatal, midpalatal, and distopalatal. Alveolar bone loss data represent the mean of the six measured sites in millimeters. The furcation of the maxillary first molar was also analyzed for bone volume density (BV/TV), bone mineral density (BMD), trabecular number (Tb.N), trabecular separation (Tb.Sp), and trabecular thickness (Tb.Th).

### Histological and histomorphometric analyses

Maxillary specimens were fixed in 4% paraformaldehyde, decalcified in 10% EDTA (Beyotime Biotechnology, Shanghai, China) for 4 weeks, and paraffin-embedded. Decalcified tissues were sectioned coronally (5 µm thickness) through the mesiodistal plane. Sections underwent hematoxylin and eosin (H&E; Beyotime Biotechnology) staining for general histological assessment.

For osteoclast identification, tartrate-resistant acid phosphatase (TRAP) staining was performed using a commercial kit (Servicebio, Wuhan, China) following the manufacturer’s protocol. TRAP-positive multinucleated osteoclasts were quantified within the alveolar bone region adjacent to the maxillary first molar using light microscopy. Histomorphometric analysis, including calculation of the osteoclast-positive area percentage, was conducted using ImageJ software.

### Immunohistochemical and Immunofluorescence Staining

Sections were blocked with 5% bovine serum albumin (BSA)/PBS for 30 min at room temperature to minimize non-specific binding. Primary antibodies targeting osteogenic markers [osteocalcin (OCN; 1:200); Servicebio, Wuhan, China] were applied and incubated overnight at 4°C. After PBS washes, sections were incubated with a biotin-conjugated secondary antibody (1:500) for 1 h at room temperature. Antigen visualization was achieved using a streptavidin-biotin-peroxidase complex with 3,3’-diaminobenzidine (DAB) as the chromogen.

To evaluate macrophage polarization markers and cytokine expression, sections were blocked with 5% BSA/PBS for 1 h at room temperature. Primary antibodies against iNOS (M1 marker), Arg-1 (M2 marker), interleukin-6 (IL-6), and interleukin-10 (IL-10) (all from Servicebio, Wuhan, China) were incubated overnight at 4°C. Following PBS washes, sections were incubated for 1 h at room temperature with species-appropriate secondary antibodies conjugated to FITC or CY3 (Servicebio, Wuhan, China). Nuclei were counterstained with 4’,6-diamidino-2-phenylindole (DAPI). Fluorescence images were acquired using a fluorescence microscope.

### Statistical analysis

Data are expressed as mean ± standard deviation (SD). Normality was assessed using the Shapiro-Wilk test. When comparing continuous variables across more than two independent groups, Analysis of Variance (ANOVA) was applied if normality was confirmed, and the Kruskal-Wallis test if not. For post-hoc intergroup comparisons following one-way ANOVA, Tukey’s test was used. Statistical analyses were performed using GraphPad Prism v8.0. Statistical significance was defined as P<0.05.

## Results

### PNS alleviates alveolar bone loss in diabetic periodontitis rats

**Figure 1:**
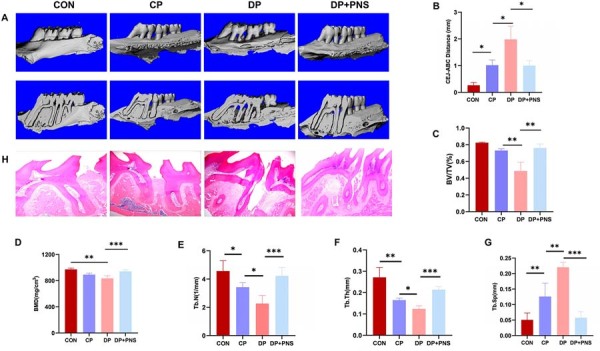
PNS ameliorates alveolar bone loss in diabetic periodontitis rats. (A) Representative micro-CT images of alveolar bone resorption in the control group, periodontitis group (CP), diabetic periodontitis group (DP), and diabetic periodontitis group treated with PNS (DP + PNS ). (B) Vertical distances between the alveolar bone crest (ABC) and the cementoenamel junction (CEJ). (C-G) Quantitative bar charts depicting BV/TV (%), BMD, Tb.Th, Tb.N, and Tb.Sp in different groups. (H) H&E staining. *P<0.05, **P<0.01,***P<0.001.

Alveolar bone height loss was quantified by measuring the vertical distance between the cementoenamel junction (CEJ) and alveolar bone crest (ABC) at the maxillary first molar. Micro-computed tomography (Micro-CT) analysis revealed a significantly greater CEJ-ABC distance in the diabetic periodontitis (DP) group compared to both the control and periodontitis control (CP) groups, whereas PNS treatment decreased CEJ-ABC distance compared to the DP group (Figure 1A, 1B). Moreover, analysis of the furcation region revealed severe impairment of bone microstructure in the DP group, characterized by significant decreases in bone volume fraction (BV/TV), bone mineral density (BMD), trabecular number (Tb.N), and trabecular thickness (Tb.Th), alongside a marked increase in trabecular separation (Tb.Sp) (Figure 1C-1G). Treatment with PNS effectively alleviated these pathological changes. Compared with the DP group, PNS significantly increased BV/TV, BMD, Tb.Th, and Tb.N, while reducing Tb.Sp (Figure 1C-1G).

Histologically, H&E staining confirmed extensive periodontal tissue destruction, including alveolar bone resorption and periodontal ligament fiber loss, in the DP group. PNS administration markedly reduced this tissue damage, leading to improved structural integrity (Figure 1H).

### PNS suppresses osteoclastogenesis in diabetic periodontitis rats

**Figure 2:**
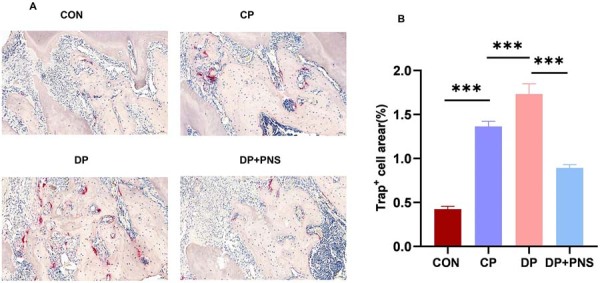
PNS suppresses osteoclastogenesis in diabetic periodontitis rats. (A) TRAP staining images in different groups. Scale bars: 100µm. (B) Percentage of osteoclast-positive area in different groups. ***P<0.001.

TRAP staining demonstrated a significant increase in the percentage of osteoclast-positive area within the alveolar bone adjacent to the maxillary first molar in the DP group relative to the control and CP groups. PNS treatment effectively reduced osteoclast-positive area (Figure 2).

### PNS upregulates osteogenic marker expression in diabetic periodontitis rats

**Figure 3:**
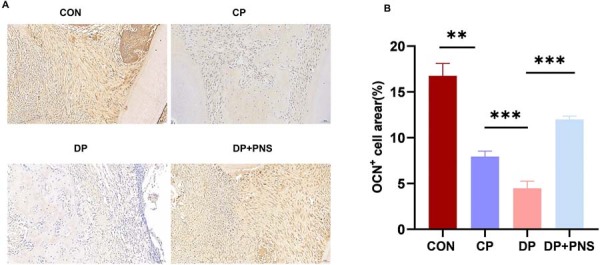
PNS promotes osteoblast-associated protein expression in diabetic periodontitis rats. (A) Immunohistochemical images of OCN in different groups. Scale bars: 50µm. (B) Percentage of OCN+ area in different groups. **P<0.01, ***P<0.001.

Immunohistochemical analysis of periodontal tissues revealed significantly enhanced protein expression levels of osteocalcin in the PNS-treated group compared to the DP group (Figure 3). This upregulation of key osteogenic markers suggests that PNS promotes bone regeneration in diabetic periodontitis.

### PNS reprograms macrophage phenotypes from M1 to M2 in diabetic periodontitis rats

**Figure 4:**
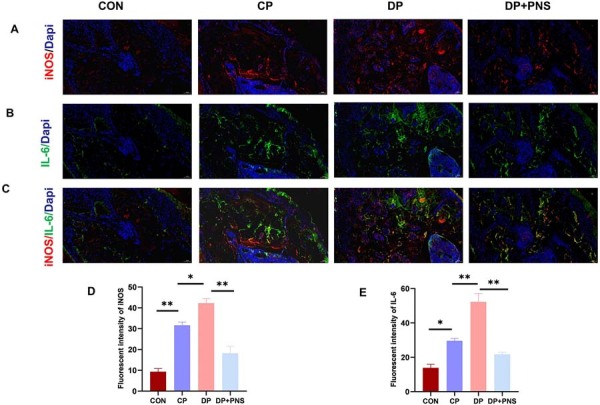
PNS decreases the expression of the M1 macrophage marker iNOS and the pro-inflammatory cytokine IL-6 in diabetic periodontitis rats. (A) Immunofluorescence staining images of iNOS in different groups. Scale bars: 50µm. (B) Immunofluorescence staining images of IL-6 in different groups. Scale bars: 50µm. (C) Merged images of iNOS/IL-6 in different groups. Scale bars: 50µm. (D) Quantitative analysis of iNOS fluorescence intensity. (E) Quantitative analysis of IL-6 fluorescence intensity. *P<0.05, **P<0.01.

**Figure 5:**
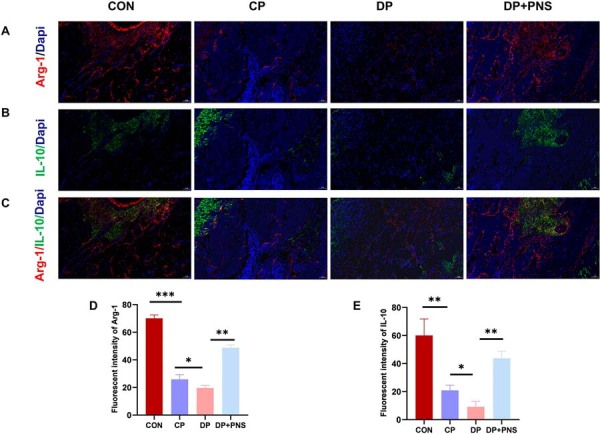
PNS increases the expression of the M2 macrophage marker Arg-1 and the anti-inflammatory cytokine IL-10 in diabetic periodontitis rats. (A) Immunofluorescence staining images of Arg-1 in different groups. Scale bars: 50µm. (B) Immunofluorescence staining images of IL-10 in different groups. Scale bars: 50µm. (C) Merged images of Arg-1/IL-10 in different groups. Scale bars: 50µm. (D) Quantitative analysis of Arg-1 fluorescence intensity. (E) Quantitative analysis of IL-10 fluorescence intensity. *P<0.05, **P<0.01, ***P<0.001.

Immunofluorescence staining indicated a significant increase in expression of the M1 macrophage marker iNOS and a decrease in the M2 marker Arg-1 within periodontal tissues of the DP group compared with control and CP groups. PNS treatment reversed this polarization imbalance, significantly reducing iNOS expression while elevating Arg-1 expression. Furthermore, PNS administration decreased levels of the pro-inflammatory cytokine IL-6 and increased expression of the anti-inflammatory cytokine IL-10 (Figures 4 and 5).

## Discussion

Diabetic periodontitis (DP) is a common complication of diabetes, characterized by progressive alveolar bone loss and periodontal tissue destruction. Its core pathological mechanisms involve hyperactive osteoclasts, inhibited osteogenic differentiation, and imbalanced macrophage phenotypes.^10,25,26^ This study demonstrated via multi-dimensional experiments that PNS effectively alleviates alveolar bone loss in diabetic rats by regulating bone metabolism balance, inhibiting osteoclastogenesis, promoting osteogenic differentiation, and reprogramming macrophage phenotypes—thus providing a new potential target for the clinical treatment of DP.

As a chronic metabolic disorder characterized by persistent hyperglycemia, diabetes mellitus (DM) significantly exacerbates periodontal destruction by disrupting bone homeostasis and amplifying inflammatory responses. Preclinical studies using diabetic animal models have consistently demonstrated accelerated alveolar bone resorption, corroborated by clinical observations of impaired bone remodeling in diabetic periodontitis patients.^5,13^Our experimental data align with these established patterns, as evidenced by micro-CT quantification and histopathological analysis (H&E staining) in the DP group. However, PNS treatment markedly reversed these pathological changes, with the PNS group showing improved bone microstructure, reduced periodontal tissue damage, and preserved alveolar bone integrity.

OCN, a late-stage osteogenic differentiation marker, is essential for matrix mineralization. Our immunohistochemical findings demonstrate that PNS significantly upregulates OCN expression in the periodontal tissues of diabetic periodontitis (DP) rats compared with untreated DP controls. This upregulation in the PNS group strongly suggests enhanced osteogenic differentiation and functional activation of periodontal ligament stem cells (PDLSCs). The observed increase in osteogenic markers correlates with PNS-induced histological improvements in alveolar bone, supporting its role in restoring periodontal tissue homeostasis. These findings strongly indicate that PNS exerts a protective effect on alveolar bone under diabetic conditions, with its pro-osteogenic activity likely being a central mechanism.

*In vitro*, the bone quality enhancing effects of PNS and its constituents may be attributed to regulation of multiple signaling pathways, including Wnt/β-catenin, BMP/BMP-R, AMPK/mTOR, and GPER/PI3K/AKT.^22^ Furthermore, PNS can enhance angiogenesis via VEGF secretion, which may indirectly support osteogenesis by improving blood supply to bone tissues, a critical factor in fracture healing.^18^

In diabetic periodontitis, excessive osteoclast activation constitutes a pivotal pathological mechanism driving alveolar bone resorption. In our study, therapeutic intervention with PNS significantly attenuated pathological osteoclastogenesis, as quantified by TRAP staining. Mechanistically, recent evidence suggests that PNS suppresses osteoclast differentiation via multiple regulatory pathways. By blocking IκBα degradation and ERK/JNK phosphorylation, PNS interrupts NF-κB and MAPK pathway activation, as demonstrated in a study by Peng. et al.^27^ This direct interference with key signaling cascades curtails the osteoclastogenic process. Furthermore, PNS restores the physiological RANKL/OPG ratio, creating an anti-resorptive microenvironment.^23^ The modulation of this crucial ratio tilts the balance toward reduced bone resorption. Additionally, PNS also scavenges reactive oxygen species (ROS) through Nrf2/HO-1 pathway activation,^28^ effectively neutralizing diabetes-enhanced oxidative stress that normally promotes osteoclast precursor differentiation. Such comprehensive regulation of osteoclast-related processes positions PNS as a potential therapeutic agent for conditions associated with abnormal bone resorption, considering its capacity to influence multiple key pathways in osteoclast biology.

Macrophage polarization critically influences periodontitis progression: pro-inflammatory M1 macrophages exacerbate bone resorption through IL-6 and TNF-α secretion, whereas anti-inflammatory M2 macrophages facilitate tissue repair via cytokines such as IL-10. Our immunofluorescence analysis demonstrated that the DP group exhibited significantly elevated expression of the M1 marker iNOS alongside reduced expression of the M2 marker Arg-1. This polarization shift was accompanied by upregulated IL-6 and downregulated IL-10, collectively indicating a pro-inflammatory milieu dominated by M1 macrophages in diabetic periodontal tissues. Notably, PNS treatment reversed this immune imbalance, promoting M1-to-M2 phenotype conversion and suppressing local hyperinflammation. This aligns with a study demonstrating PNS-mediated M2 polarization in THP-1 cell models under hyperglycemic conditions by downregulating the NF-κB signaling pathway.^29^ Similarly, PNS promotes STAT6-dependent M2 polarization in LPS-induced acute lung injury, leading to increased IL-10 secretion and reduced neutrophil infiltration.^30^ These findings highlight PNS’s capacity to orchestrate macrophage behavior via direct signaling mechanisms.

## Conclusions

In conclusion, this study demonstrates that PNS effectively attenuates alveolar bone loss in diabetic rats via a triple-action mechanism: suppression of osteoclastogenesis, promotion of osteogenic differentiation, and reprogramming of macrophage phenotypes. These findings establish a pharmacological basis for targeting DP and identify potential therapeutic nodes. However, this study remains limited to *in vivo* experiments, investigating the role of PNS in whole animal models. Consequently, *in vitro* experiments have not yet been conducted to elucidate the direct or indirect effects of PNS. In future studies, we plan to include relevant *in vitro* validations along with mechanistic analyses.
